# Glow or No‐Go: Ethical considerations of adolescent and teen skincare trends in social media

**DOI:** 10.1111/srt.70029

**Published:** 2024-08-26

**Authors:** Aarushi K. Parikh, Shari R. Lipner

**Affiliations:** ^1^ Rutgers Robert Wood Johnson Medical School New Brunswick New Jersey USA; ^2^ Department of Dermatology Weill Cornell Medicine New York New York USA

Dear Editor:

Increased access to social media applications has dramatically shifted how teens and adolescents engage with beauty and skincare trends. Many trending skincare routines feature ingredients, which may not be beneficial and cause adverse reactions.^1^ Risks and concerns include increased sun damage, irritation, inflammation, scarring, and psychological impacts from an obsession with “perfect skin” from a young age.^2^ Reports of adolescents and teens experiencing adverse effects after trying beauty trends endorsed by social media influencers^3^ demonstrate the potential consequences of following trends without sufficient understanding of their safety and suitability. Although adolescents and teens can make adult‐level decisions, they frequently do not exercise it optimally, leading to more immediate‐reward decisions enhanced by peers.^4^

While adolescents and teens may assert autonomy in experimenting with skincare routines, they may not have decisional capacity^5^ to make informed decisions about these products. The ethical responsibility lies in ensuring they have access to accurate information and guidance when using such products. Parents and guardians play a pivotal role in balancing protection of their children with fostering autonomy and independence. Given growing interest in skincare, adolescents should be encouraged to speak with a dermatologist who can provide age‐appropriate skin recommendations that promote long‐term skin health.

However, youth are constantly exposed to persuasive marketing and peer‐driven beauty trends that may not prioritize their long‐term well‐being over immediate aesthetic appeal. They desire to emulate the skincare regimens of their favorite influencers, driven by the allure of luxury products perceived as essential for achieving flawless skin. Ethical skincare practices must advocate for transparency in marketing, promoting evidence‐based approaches that prioritize safety and health over superficial trends to avoid harm to young individuals. Thus, influencers and content creators also have an obligation of transparency about their target audiences and mitigating the risk of exploiting youthful impressionability for commercial gain, especially when promoting skincare products knowingly to younger viewers. Influencers hold significant sway over adolescent audiences, influencing their perceptions of beauty ideals and skincare routines. As a creator's viewership expands, so does their duty to promote truthful practices.

Some may argue parents or guardians have the responsibility of regulating purchases and skincare exploration. However, the pervasive influence of social media exposes youth to content through their peers, educational institutions, and various other channels where social interactions and commercial interests converge. This extensive reach complicates the traditional notion of parental responsibility.

Ultimately, the solution lies in empowering adolescents and teens with the knowledge and critical thinking skills to navigate skincare choices responsibly. While caregivers play a role, influencers and dermatologists equally can foster an informed approach to adolescent skincare practices in the digital age. Discussion about media literacy must be initiated to help young individuals discern between genuine skincare needs and aspirational marketing. Influencers must emphasize realistic expectations and effective skincare routines beyond lavish products. Physicians should advocate for the promotion of affordable skincare alternatives that cater to different economic backgrounds. Collaborative efforts are required to protect adolescent and teen health and cultivate the necessary foundation to navigate skincare choices wisely.

## CONFLICT OF INTEREST STATEMENT

The authors declare no conflicts of interest.

## Data Availability

Data sharing not applicable to this article, as no datasets were generated or analyzed during the current study.

## References

[1] Kids into skincare may be at risk from influencers promoting inappropriate products. Uclahealth.org. Accessed June 21, 2024. Published January 25, 2024. https://www.uclahealth.org/news/article/kids‐skincare‐dermatologists‐advice

[2] Rodan K , Fields K , Majewski G , Falla T . Skincare bootcamp: the evolving role of skincare. Plast Reconstr Surg Glob Open. 2016;4(12 Suppl):e1152. 28018771 10.1097/GOX.0000000000001152 PMC5172479

[3] McCoy K , Class MM , Ricles V , et al. Kids these days: social media's influence on adolescent behaviors. J Clin Aesthet Dermatol. 2024;17(5):40‐42. 38779370 PMC11107899

[4] Diekema DS . Adolescent brain development and medical decision‐making. Pediatrics. 2020;146(Supplement 1):S18‐S24. doi:10.1542/peds.2020-0818f 32737228

[5] Hawkins J , Charland LC . Decision‐Making Capacity (Stanford Encyclopedia of Philosophy). Stanford.edu. . Accessed June 21, 2024. Published 2020. https://plato.stanford.edu/entries/decision‐capacity/#CompCapa

